# Maternal undernutrition in the first eighty days of gestation negatively programs ovarian development in dairy calves^†^

**DOI:** 10.1093/biolre/ioae158

**Published:** 2024-11-07

**Authors:** Adele Frau, David Edache, Sebastiano Sale, Antonio Gallo, Vincenzo Miragliotta, Giulia Lazzarini, Andrea Corda, Francesca Corda, Olimpia Barbato, Sara Succu, Daniela Bebbere, Federica Franciosi, Alberto S Atzori, Francesca Mossa

**Affiliations:** Department of Veterinary Medicine, University of Sassari, Sassari, Italy; Department of Agriculture, University of Sassari, Sassari, Italy; Veterinary practitioner, Dorgali (NU), Italy; Department of Animal Science, Food and Nutrition, Università Cattolica del Sacro Cuore, Milan, Italy; Department of Veterinary Medicine, University of Pisa, Pisa, Italy; Department of Veterinary Medicine, University of Pisa, Pisa, Italy; Department of Veterinary Medicine, University of Sassari, Sassari, Italy; Department of Veterinary Medicine, University of Sassari, Sassari, Italy; Department of Veterinary Medicine, University of Perugia, Perugia, Italy; Department of Veterinary Medicine, University of Sassari, Sassari, Italy; Department of Veterinary Medicine, University of Sassari, Sassari, Italy; Department of Veterinary Medicine, University of Milan, Lodi, Italy; Department of Agriculture, University of Sassari, Sassari, Italy; Department of Veterinary Medicine, University of Sassari, Sassari, Italy

**Keywords:** Developmental Origins of Health and Disease, leptin, testosterone, pregnancy, undernutrition, ruminants, anti-Müllerian hormone, ovarian reserve, heart, arterial blood pressure

## Abstract

We hypothesized that in dairy cattle maternal energy restriction applied during two gestational windows (up to day 80 or 120 of gestation) impairs ovarian and cardiovascular development in juvenile female offspring. We also investigated the role of maternal leptin and testosterone in developmental programming in calves. Holstein-Friesian heifers were randomly assigned to one of three experimental groups; starting 10 days before artificial insemination, they were individually fed at (i) 0.6 of their maintenance energy requirements (M) up to day 80 (Nutrient Restricted, NR80) or (ii) day 120 of gestation (NR120); (iii) 1.8 M until day 120 of pregnancy (Control). Plasma leptin concentrations increased transiently in nutritionally restricted heifers pregnant with a single female calf, but maternal testosterone concentrations were not influenced by diet. Calves had similar body growth, but daughters of NR80 and NR120 had impaired ovarian development, as assessed by reduced gonadal weight, fewer surface antral and primary follicles, and recovered cumulus-oocyte complexes, as well as lower circulating anti-Müllerian hormone concentrations. Cardiovascular morphology and function in the offspring were not influenced by maternal diet, as determined by peripheral arterial blood pressure, echocardiography, *post-mortem* heart weight, and aortic circumference. Regardless of its duration (until day 80 or 120 of gestation), nutritional restriction resulted in a similar alteration of ovarian development in juvenile progeny, but cardiovascular development was unaltered. Evidence suggests that the window of development that encompasses the peri-ovulatory period to the first 2.6 months of gestation is critical in ovarian programming and that maternal leptin may be involved.

## Teaser

Energy restriction to day 80 or 120 of gestation enhanced maternal leptin, decreased serum anti-Müllerian hormone, gonadal weight, surface antral and primary follicles, recovered cumulus-oocyte complexes, but had no impact on body weight and cardiovascular system in dairy calves.

## Graphical Abstract

**Graphical Abstract:**
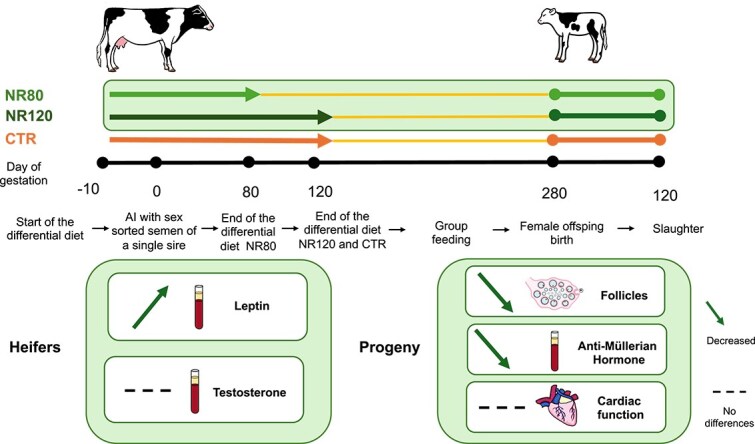

## Introduction

Early environmental factors during fetal development can exert a profound influence on postnatal health and disease outcomes in mammals [1]. Thus, controlling gestational conditions as a means to induce beneficial effects or reduce potential negative impacts on the offspring represents a novel and promising field of research. The Barker hypothesis, rooted in the field of Developmental Origins of Health and Disease, provides a compelling perspective on how early life experiences, particularly during fetal development, can significantly impact health in later life [2–5]. Notably, evidence suggests that various factors acting during gestation can influence the future reproductive performance of female offspring in ruminants [6].

The bovine species is one of the most suitable experimental models to investigate folliculogenesis and ovarian follicular dynamics in mammals [7, 8]. In cattle, ovarian follicular growth takes around 3–4 months and consists of a gonadotrophin-independent stage followed by a gonadotrophin-dependent phase [9]. The gonadotrophin-independent phase starts with the activation of primordial follicles that leave the resting pool, grow to primary follicles, and subsequently into preantral follicles [10, 11]. The gonadotrophin-dependent phase is characterized by follicle-stimulating hormone (FSH)-induced waves of antral follicle growth [12–14].

The total number of healthy oocytes and follicles in the ovaries, named ovarian reserve, is established during fetal life in cattle [15]. Several studies revealed that the most critical period for the establishment of the ovarian reserve corresponds to early to mid-gestation phase in cattle. Indeed, primordial follicle formation in cattle was described at 74 [16], 90 [17, 18], and 110 days of fetal life [19]. During fetal life, follicular development is associated with the degeneration of ovarian germ cells [15]. Evidence shows that the peak number of germ cells occurs between days 91 and 110 in cattle, reaching an estimated maximum number of 2.1 million [20]. However, from this peak, 80–90% of germ cells undergo apoptosis [11]. Simultaneously, the rate of germ cell proliferation decreases, and the number of healthy follicles and oocytes present at birth varies widely, ranging from 10 000 to 350 000 in cattle [21, 22].

Peripheral concentrations of anti-Müllerian hormone (AMH) have been validated as *in vivo* indicators of the size of the ovarian reserve size in cattle [23]. The AMH is a glycoprotein hormone produced by the granulosa cells in growing ovarian follicles [23] both in ruminants and women [24–27]; AMH is not generated by primordial or atretic follicles, but its production commences upon the recruitment of follicles [28]. Peak AMH secretion is observed during the preantral and small antral follicle stages, subsequently decreasing as the selected follicles mature to the preovulatory stage under FSH influence [23, 29]. Serum AMH concentrations exhibit considerable variability among individuals but demonstrate high repeatability within the same animal [30]. Furthermore, there is compelling evidence supporting a strong positive correlation between AMH serum levels in age-matched individuals and the antral follicle population in various species, such as cattle [31–33] and small ruminants [34].

Scientific findings suggest that prenatal exposure to energy restriction may influence the growth and physiological function of the female reproductive system. Previous evidence from our groups showed that providing an energy-restricted diet to beef heifers (0.6 of their estimated maintenance energy requirements, M) from 10 days before conception (CR) to the first trimester of pregnancy (110 days of gestation; DG) had a detrimental impact on the size of the ovarian follicular reserve of their daughters after birth [35]. Despite body weight (BW) being unaltered from birth to adulthood, the number of antral follicles recruited per follicular wave and AMH were lower in female progeny born to mothers on a restricted diet compared to daughters born to control dams [35]. Similarly, pregnant ewes fed a low (50% M) compared to high energy (150% M) diet from mating to early gestation, had daughters with different fetal ovarian development. Reduced numbers of oogonia were detected at DG 47 in fetuses exposed to high compared to low maternal energy diet, but at DG 62, the degeneration of germ cells was reduced in low energy than high energy offspring [36]. Ewes born to dams underfed from mating to DG 95 had reduced ovulation rates compared to offspring of control dams, resulting in lower reproductive performances [37]. These findings provide evidence for a detrimental impact of energy imbalance *in utero* on the development of the female gonads, yet the mechanisms whereby transient nutrient restriction may influence ovarian development remain unclear, and the most sensitive windows of development have not been clearly identified.

Maternal nutrient restriction has also been associated with increased androgen peripheral concentrations in pregnant heifers. We observed higher circulating maternal testosterone levels in underfed beef heifers compared to dams fed a control diet during the initial 110 DG [35]. Prenatal exposure to testosterone excess has been extensively investigated using an ovine model of polycystic ovarian syndrome [38, 39]. When ewes are exposed to exogenous testosterone from the beginning of gonadal differentiation to shortly after the formation of primordial follicles (from DG 30 to 90), their ovarian reserve is reduced [40], and antral follicles accumulate, potentially because of increased follicular recruitment [41] or by an increased follicular persistence [42]. Maternal androgens also play a role in cardiovascular system differentiation, function, and pathology of the progeny [43]. Androgen receptors were detected in fetal cardiovascular system [44] and prenatal testosterone excess may induce hypertension in female sheep [45]. We reported the enlargement of the aorta and increased peripheral arterial blood pressure (BP) in female beef cattle born to nutritionally restricted mothers compared with controls [35]. Given the common embryonic precursor (aortic-gonadal mesonephros) and the presence of androgen receptors in both cardiovascular and reproductive systems [46], it is plausible that maternal nutrient restriction may result in increased androgen concentrations in the dams, which in turn may influence the development of the ovaries and of the cardiovascular system in their progeny. Leptin, a peptidic metabolic hormone produced by adipose tissue, may play a crucial role in testosterone regulation by modulating the secretion of gonadotrophin-releasing hormone (GnRH) [47, 48].

In the present work, we hypothesized that maternal energy restriction, initiated from 10 days before CR and applied during two gestational windows (up to DG 80 or 120), may impair the development of ovaries and the postnatal function of the cardiovascular system in female offspring of dairy cattle. Moreover, we aimed at investigating the potential role of maternal leptin and testosterone circulating concentrations in developmental programming in dairy calves.

## Materials and methods

All animal experiments were performed in accordance with DPR 27 January 1992 (Animal Protection Regulations of Italy) in conformity with European Community regulation 86/609 and were approved by the local Committee for the Animal Welfare of the University of Sassari (prot. n. 0001848, 2 May 2019).

### Animals, feeding regimen, estrus synchronization, and artificial insemination

This study was conducted from April 2021 to July 2022 in a commercial dairy farm located in North Sardinia, Italy (40°35′29.8” N, 8°53′19.9″ E). The herd consisted of 650 Holstein-Friesian cattle, 310 of which were lactating cows. The mean milk production per animal/day was 35 kg, for a total amount of 11.500 liters per year (Composition: 4.20% lipids; 3.45% proteins). Adult Holstein-Friesian nulliparous heifers were enrolled (*n* = 42; BW: 366.2 ± 41.1 kg; age: 16.2 ± 1.22 months.; mean ± SD) and were randomly assigned to one of the following experimental groups, balanced on BW and age: Nutrient Restricted (NR) (*n* = 32; BW = 366.1 ± 42.67 kg; age = 16.1 ± 1.13 months) and Control, CTR (*n* = 10; BW = 368.8 ± 39.1 kg; age = 16.34 ± 1.55 months); NR heifers were assigned a diet providing 0.6 of their estimated maintenance energy requirements (M), whereas CTR animals were fed at 1.8 M. All heifers were located in paddocks with self-capturing racks and individually fed twice daily (08:00 and 16:00 h), starting 10 days before artificial insemination (AI). The diet was a total mixed ration (TMR) formulated based on the energetic intake of the diet. The ration consisted of a commercial complete feed *(FiberFeed®*, Cooperativa Produttori Arborea, Italy). Diet (Table 1) was prepared using the Large Ruminant Nutrition System with modifications from the equations developed by Fox [49]. Dry TMR contained 34.5% grass hay, 19.2% steam-flaked corn, 3.9% cane-beet molasses blend, and 42.4% grain mix (29.6% wheat bran, 29.4% sorghum grain, 21.6% soybean meal, 14.7% flaked soybean, 2.2% calcium carbonate, 1% sodium chloride, 0.4% magnesium oxide, 0.9% sodium bentonite), and 0.3% vitamin and mineral premix that provided 40 000 IU of vitamin A, 4000 IU of vitamin D3, 30 mg of vitamin E 92% α-tocopherol, 5 mg of vitamin B1, 3 mg of vitamin B2, 1.5 mg of vitamin B6, 0.06 mg of vitamin B12, 5 mg of vitamin K, 5 mg of vitamin H1 (para-aminobenzoic acid), 150 mg of vitamin PP (niacin), 50 mg of choline chloride, 100 mg of Fe, 1 mg of Co, 5 mg of I, 120 mg of Mn, 10 mg of Cu, and 130 mg of Zn. All heifers received two injections of PGF_2α_ (Cloprostenol, PGFVeyx™, Bayer; 2 mL IM) spaced 11 days apart and, starting 12 h after the second injection, were visually monitored to detect signs of estrus and the presence of a preovulatory follicle (PO) was assessed via reproductive ultrasonography (MyLab™Omega equipped with a 4–10 MHz linear transrectal probe, Esaote, Italy). Approximately 8 h after standing heat was observed, heifers were artificially inseminated with sex-sorted semen from a single sire (Barbaro, *InSeme,* Italy) to reduce paternal impact in the progeny and to increase the number of female calves born. Pregnancy was diagnosed at approximately 28 days post-AI, and fetal sex was determined at DG 60 via reproductive ultrasonography. Non-pregnant heifers and dams pregnant with a male calf were excluded from the study and returned to the herd. Sixty days post-AI, NR heifers pregnant with a single female calf (*n* = 19) were divided into two groups, balanced on body weight: NR80 (*n* = 9; BW = 388.5 ± 14.5 kg) and NR120 group (*n* = 10; BW = 404.3 ± 11.6 kg), which were individually fed the NR diet (0.6 M) up to DG 80 and 120, respectively. Heifers pregnant with a single female calf in the CTR group (*n* = 5) were individually fed at 1.8 M until DG 120. After the end of the differential feeding regime, all heifers were housed together and had *ad libitum* free access to wet TMR until calving. Wet TMR (kg/DM) contained 4.01 kg ryegrass hay, 2.40 kg of ryegrass silage, 0.43 kg of ground fine corn, 0.88 kg soybean meal 48%, 0.10 kg 4020 optimizer complex, and 2.18 kg mineral premix (Table 1).

**Table 1:** Chemical composition (% DM), particle size distribution on an as-fed basis, and diet physically effective neutral detergent fiber (peNDF) of the TMR offered to the dams. During the differential feeding, the TMR was individually offered from 10 d before AI to day 80 to NR80 dams and to day 120 of gestation to NR120 and CTR heifers, respectively. From day 81 (NR80) and day 121 of gestation (NR120 and CTR) to calving, dams were group fed *ad libitum* the postdifferential feeding TMR

Item	Dry TMR (differential feeding)	Wet TMR (postdifferential feeding)
Dry matter, DM % as fed	96.6	95.2
Crude protein, CP (% DM)	14.5	12
Neutral detergent fiber, NDF (% DM)	45.3	48.7
Acid detergent fiber, ADF (% DM)	30.2	32.6
Ether extract, EE (% DM)	1.4	2.5
Acid detergent lignin, ADL (% DM)	4.3	4.2
Ash (% DM)	9.7	9.0
Acid detergent insoluble protein, ADIP (% DM)	1.0	0.9
Sugars (% DM)	5.2	4.7
Soluble protein, SOLP (% DM)	4.2	4.9
Neutral detergent insoluble protein, NDIP (% DM)	3.2	1.8
Starch (% DM)	16.3	11.9
Nonfiber carbohydrates, NFC (% DM)^1^	29.1	28.9
Metabolizable energy, ME^2^ (Mcal/kg)	2.28	2.26
Penn State Particle Separator, PSPS (Mean ± SD)		
19 mm (%)	12.9 ± 1.99	28.3 ± 9.09
8 mm (%)	26.9 ± 4.29	42.4 ± 7.77
4 mm (%)	19.1 ± 0.99	14.9 ± 1.1
Bottom (%)	41.2 ± 2.67	14.4 ± 4.13
Physically effective NDF, peNDF	27.44	31.60

^1^Calculated as NFC = 100 − CP − ash − NDF − ether extract.

^2^Calculated using NRC, 2001 guidelines.

Dry matter value for the wet TMR was computed after samples were dried in the oven at 65°C for 24 h.

Dry TMR contained 34.5% grass hay, 19.2% steam-flaked corn, 3.9% cane-beet molasses blend, and 42.4% grain mix (29.6% wheat bran, 29.4% sorghum grain, 21.6% soybean meal, 14.7% flaked soybean, 2.2% calcium carbonate, 1% sodium chloride, 0.4% magnesium oxide, 0.9% sodium bentonite), and 0.3% vitamin and mineral premix. Wet TMR (kg/DM) contained 4.01 kg ryegrass hay, 2.40 kg of ryegrass silage, 0.43 kg of ground fine corn, 0.88 kg soybean meal 48%, 0.10 kg 4020 optimizer complex, and 2.18 kg mineral premix.

During the individual feeding phase daily metabolizable energy intake (MEI) was determined as the metabolizable energy (ME) in the TMR offered minus ME in the orts on a daily basis, while MEI post differential feeding was determined as the ME in the feed multiplied by the dry matter intake (DMI) determined by prediction equations. DMI at the end of the individual feeding period was calculated based on following predicted equations [50, 51].

1. DMI = 12.91 × (1 − e(−0.00295 × BW) [[51]; end of each individual feeding to day 250 of gestation].

2. DMI = (1.71 − (0.69× eˆ((0.35 × DP − 280))))/100 × BW [[50]1; >250 to calving] where e is the Euler number (e = 2.718), BW is the body weight, and DP is the day in pregnancy.

### Assessment of the size of the preovulatory follicle and of antral follicle count of the dams

Ultrasonographic exams of the reproductive tracts were conducted on three occasions: on the day of AI, DG 28, and DG 55–65; videos were recorded and analyzed with Windows 10® by a single operator, who was blinded by the heifer ID. The area of the PO and the ovarian area were estimated in heifers that showed estrous behavior (*n* = 40; NR *n* = 31; CTR *n* = 9) by measuring the two largest internal diameters in two perpendicular planes on the day of AI and by using the ellipse formula (Area = πab, cm^2^). The total number of antral follicles ≥3 mm in diameter (antral follicular count, AFC) was estimated on the day of AI (*n* = 40; CTR *n* = 9; NR *n* = 31) and on DG 28 and 55–65 (*n* = 28; NR *n* = 22; CTR *n* = 6), as previously described [52–54].

### Maternal body weight, ADG, and circulating leptin and testosterone concentrations during pregnancy

All heifers were weighed monthly with a digital scale fitted in a manual chute. To determine the impact of nutrient restriction on circulating concentrations of leptin and testosterone, blood samples were collected from heifers during gestation on a monthly basis, starting 11 days prior to AI. Average daily gain (ADG, kg/day) was calculated by dividing the difference in BW between pre-CR and DG by the number of days from pre-CR to the DG. Blood was collected from the coccygeal vein, using 20G needles with the vacutainer system red cap (added with Clot activator) and purple cap (added with K3EDTA) 9 mL tubes (Vacutest®, Kima). Each tube was immediately refrigerated and transported to the laboratory.

### Body weight, biometric measurements, and circulating concentrations of AMH in the offspring

Twenty-four single female calves were born (CTR *n* = 5; NR120 *n* = 10; NR80 *n* = 9). One NR80 calf and her dam were retrospectively excluded from the study because the calf had a pathologically heavier and larger ovary compared to the herd-mates. One NR120 calf died when she was 30 days old due to omphalitis; thus only data from her dam were included in the study. Calves were immediately separated from their dams and managed following the routine farm protocol. Briefly, calves were administered colostrum within 6 h after birth and for the first 4 days of life. Calves were gradually fed increasing amount of milk replacer (Servatec, Excell) from day 5 to 60 of age. From day 60 to 70, milk replacer administration decreased progressively, while the started feed (Purina®) increased from 4 to 5 kg/day. Calves growth was monitored at birth and every 2 weeks until slaughter by measuring BW and the following biometric measurements: thoracic circumference, height at withers, hip height, and back length.

To investigate the long-term impact of maternal nutrition on the size of the ovarian reserve in the progeny, blood samples were collected from the jugular vein of the calves and peripheral concentrations of AMH were measured. The sampling regime was (i) within 24 h after birth, (ii) every two weeks in the first month of life; and (iii) monthly until they were 4 months old.

### Peripheral arterial blood pressure measurement and echocardiography in the progeny

To investigate the long-term impact of maternal nutrition on the offspring’s cardiovascular system, arterial resting peripheral BP was measured in calves every fortnight starting at 30 days of age. The tail-cuff system was used with a noninvasive electronic sphygmomanometer (Cardell Veterinary Monitor 9401BP, SHARN Veterinary), previously validated in cattle [35]. The calf was gently restricted in standing position, the circumference of the base of the tail was measured, and the appropriate cuff was applied around the medial coccygeal artery at the base of the tail, based on the manufacturer’s recommendations. Systolic (SYST), diastolic (DIAST), mean (MAP) arterial BP, and heart rate (HR) were recorded 5–10 consecutive times/animal; the three readings with the lowest interreading variation (MAP variation <10 mmHg) were selected and average values calculated.

The heart morphology, dimension, and function were evaluated by serial echocardiographic examinations performed at 35 and 100 days postbirth. Echocardiography was performed by a single experienced operator with a portable ultrasound unit (My Lab Alpha, Esaote, Florence, Italy) equipped with a multifrequency (1–4 MHz) phased array transducer (SP2430). Echocardiographic examinations were performed on unsedated calves, gently restrained in a standing position. The hair coat of the right parasternal area was clipped and the transducer was placed on the clipped area in order to obtain right parasternal long- and short-axis views of the heart. B-mode, M-mode, and Doppler images and loops were stored and analyzed offline. The aortic root was visualized in B-mode from the right parasternal short-axis view at the level of the heart base. The transverse aortic diameter (Aot) was measured along the commissure between the noncoronary and right coronary valve cusps at early ventricular diastole in the first frame after aortic valve closure [55, 56]. M-mode images of the left ventricle (LV) were obtained from the right parasternal short-axis view at the level of the papillary muscles, as previously described [57]. Using M-mode images, we measured the interventricular septum in diastole (IVSd), the LV Internal diameter in diastole (LVIDd), the LV posterior wall in diastole (LVPWd), the inter-ventricular septum in systole (IVSs), the LV internal diameter in systole (LVIDs), and the LV posterior wall in systole (LVPWs). From the LVIDd and LVIDs measurements, the fractional shortening (FS%) was also obtained, as an index of LV systolic function [58]. The Doppler imaging mode was used to assess the presence of cardiac flow anomalies attributable to congenital or acquired cardiac diseases [59]. For each echocardiographic variable, three to five consecutive measurements were averaged, and the mean values were used for further statistical analyses.

### Post-mortem sampling

Calves were slaughtered in a commercial slaughterhouse when they were 19.4 weeks old (4.5 mo.). Both kidneys, the heart, and the ovaries were recovered and weighed. The internal aortic diameter and the circumference at the base of the aorta, as well as ovarian length, height, and depth were measured. Subsequently, pairs of ovaries were labeled and transported within 1–2 h to the laboratory in Dulbecco's phosphate buffered saline (DPBS) with 0.1 g/L penicillin and 0.1 g/L streptomycin) at 27–30°C. Both ovaries were washed in fresh DPBS and weighed. The visible antral follicles on the ovarian surface were counted. Cumulus-oocyte complexes (COCs) were collected and counted from each pair of ovaries. Using a micro-blade to release follicular content, the ovarian cortex was gently sliced in collection medium, prepared as 9.5 g of tissue culture medium 199 with 1 L of Milli-Q water supplemented with penicillin (0.1%) and streptomycin (0.1%), with 25 mM HEPES, 0.4 g/L bicarbonate and 0.1% (w/v) polyvinyl alcohol (PVA); pH 7.3, osmolality 290 mOsm/kg.

Following COCs collection, ovaries were fixed in 10% neutral formalin solution (pH 7.4). One ovary from each animal was embedded in 7% agar to perform Systematic Uniform Random Sampling for stereological analysis using the physical dissector method. The ovaries embedded in agar were cut into 2-mm-thick slabs using a tissue slicer. All the slabs were paraffin-embedded, and two consecutive 10-μm thick sections (the reference and the look-up sections) were collected from each slab and stained with hematoxylin and eosin. The obtained slides were acquired using the Nano Zoomer Hamamatsu slide scanner (Hamamatsu, Japan), and the primordial and primary follicles were counted in the whole images of the reference and look-up sections using Adobe Photoshop (Adobe Inc., 2019).

Primordial follicles were identified as having oocytes surrounded by flattened follicular cells, while primary follicles were identified as having oocytes surrounded by a single cuboidal layer of granulosa cells. Only healthy follicles, containing oocytes and granulosa cells showing no signs of degeneration or apoptosis, potential indicators of follicular atresia, were included in the count. The follicles seen in the reference section and not in the look-up section were counted, and then the reference and look-up sections were reversed to double the number of dissector pairs. The total number of objects counted in each slab was summed. To determine the total number of follicles in each ovary, the sum of counted follicles—either primordial or primary—was multiplied by the total inverse fractions [60]:


$$ \mathrm{N}=\frac{1}{BSF}\ \mathrm{x}\ \frac{\sum Q}{2} $$

where *ΣQ* is the total number of objects that have to be halved because of the two-directional count, and BSF is the block section fraction, calculated as the distance between the dissector pair (reference and look-up sections) divided by the distance between the slabs:


\begin{align*} BSF&=\frac{Distance\ between\ Reference\ and\ Look\ up\ sections}{Distance\ between\ the\ slabs}\nonumber\\&=\frac{10\ \mathrm{\mu} m}{2000\ \mathrm{\mu} m} \end{align*}

*Hormone analysis.*

The vacutainer tubes added with clot activator were centrifuged at 4°C at 2000 g × 15 min to obtain serum and the tubes added with K3EDTA were centrifuged at 2500 g × 15 min to retrieve plasma. Serum and plasma samples were stored in 2 mL Eppendorf tubes at −20°C until analysis.

Leptin plasmatic concentrations in the dams were measured with a commercial kit (Cattle LEP (Leptin) ELISA Kit, ELK Biotechnology, Wuhan). The analytical sensitivity was 0.061 ng/mL and the detection range was 0.16–10 ng/mL. All samples were analyzed in duplicate and the mean of the two values was calculated. If a difference greater than 10% was detected between duplicates of the same samples, samples were reanalyzed. The intra-assay coefficient of variation was 6.1%.

Serum maternal testosterone in the dams was detected using a commercial RIA kit (Ria Testosterone, Beckman Coulter, Inc© cod IM1087-01). The analytical sensitivity was 500 pg/mL and the detection range 500–20.000 pg/mL. All samples were extracted using ethyl ether before the assay, were analyzed in duplicate and the mean of the two values was calculated. The intra- and inter-assay coefficient was 11.6 and 13.5%, respectively.

Serum AMH concentrations in the progeny were measured with a commercial kit (Cattle AMH ELISA kit, ELK Biotechnology, Wuhan). The analytical sensitivity was 177.4 pg/mL and the detection range was 625–40.000 pg/mL. All samples were analyzed in duplicate and the mean of the two values was calculated and considered. If a difference greater than 10% was detected between duplicates of the same samples, samples were reanalyzed. The intra-assay coefficient of variation was 4.6%.

### Statistical analysis

All data were analyzed using R (R Core Team, 2020). The normality of the data distribution was verified using the Shapiro–Wilk test. When the data were not normally distributed, they were transformed into log base 10, but natural numbers are reported in the text. Differences in pregnancy rates (PR) between the NR and CTR heifers were analyzed with Pearson chi-square test. One NR80 calf and her dam were retrospectively excluded from the study because the calf had a pathologically heavier and larger ovary compared to the herd-mates. One NR120 calf died when she was 30 days old due to omphalitis; thus only data from her dam were included in the study. One NR 80 calf was excluded from the cardiovascular evaluation and BP measurement because an interventricular septal defect was detected at echocardiography exam. Differences in the area of the PO follicle and the repeatability of the AFC in the dams were analyzed using the LMER package (ANOVA, Type III). BW, ADG and peripheral maternal hormones (leptin, testosterone, AMH), and cardiac examination (arterial BP and echocardiography in calves) were analyzed as repeated measures within treatments using the multivariable linear regression model:


$$ Yijk=\mu + FPi+ Dj+\left( FP\ast D\right) ij+ Heifer\ (ID)k+ eijk $$

Y_ijkl_ was the independent variable; μ was the overall mean; FP_i_ was the fixed effect of gestation feeding program of pregnant heifers (three levels, CTR (NR120, NR80); D_j_ was the fixed effect of gestation day of pregnant heifers or calf age; (FR*Dj) was the interaction between the effects of FP_i_ and D_j_; heifer (ID) was the random effect of individual heifer/calf; e_ijk_ was the residual error. When the predictor effects were considered significant, the least squares means were separated using Tukey test for honestly significant differences. Post-mortem results, such as ovarian weight, volume, number of visible follicles, retrieved COCs, primordial and primary follicles, heart and kidney measures, and weight, were analyzed with one-way ANOVA and mean contrast separated with Tukey post-hoc test. Correlations between ovarian weight and primordial, primary follicles, visible antral follicles, and retrieved COCs were analyzed with Pearson correlation. Data are expressed as mean ± standard error of the means. Differences in means with *p* ≤ 0.05 were considered significant, whereas a tendency was declared when 0.05 < *p* ≤ 0.07.

## Results

### Maternal conception rates, size of preovulatory follicle, and AFC

Forty heifers (NR *n* = 31; CTR *n* = 9) showed estrous behavior in response to the estrous synchronization treatment and were artificially inseminated (Table 2). CR rates at 28 days post-AI (NR *n* = 23, 74%; CTR *n* = 6, 66%) and PR at 60 days post-AI (NR *n* = 22, 70%; CTR *n* = 6, 66%) were similar between groups. Three and one male fetuses were conceived by NR and CTR heifers, respectively.

**Table 2:** Conception and PR in dairy heifers that were individually fed a ration providing either 0.6% NR or 1.8% (CTR) of their energy maintenance needs, starting 10 days before AI. All heifers were inseminated with sex-sorted semen from a single sire; pregnancy was diagnosed by reproductive ultrasonography at approximately 28 (CR) and 60 (PR) days post-AI. At 60 days post-AI, fetal sex was also determined. No differences were detected by the Pearson chi-square test in CR and PR between NR and CTR heifers

Experimental groups	Synchronized heifers, *n*	Inseminated heifers, *n*	Pregnant heifers 28 days post-AI, *n* (CR%)	Pregnant heifers 60 days post-AI, *n* (PR%)
Total animals, *n*	42	40	29 (72%)	28 (70%)
Nutrient Restricted (NR)	32	31	23 (74%)	22^A^ (71%)
Control (CTR)	10	9	6 (66%)	6^B^ (66%)

^A^Calf sex: female *n* = 19; male *n* = 3

^B^Calf sex: female *n* = 5; male *n* = 1

**Figure 1:**
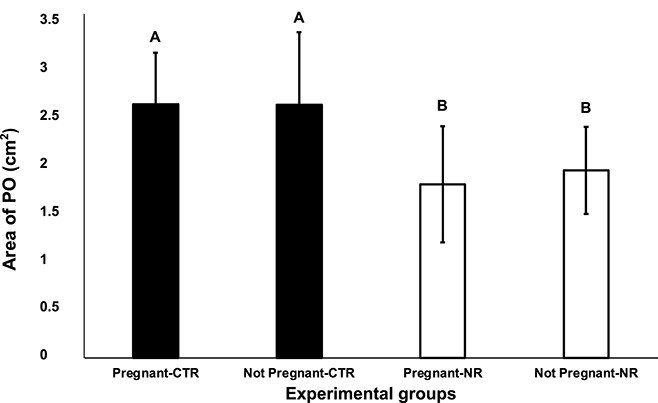
Area of the PO (cm^2^) measured via ovarian ultrasonography on the day of AI (*n* = 40; CTR n = 9; NR *n* = 31), corresponding to day 10 of the differential diet. Pregnancy was subsequently diagnosed on day 28 and 60 post-AI and heifers were retrospectively divided into: pregnant control heifers (*n* = 6); pregnant NR heifers (*n* = 22); nonpregnant control heifers (*n* = 3); and nonpregnant NR heifers (*n* = 9). The NR group was fed with a ration at 0.6% of the maintenance requirement (M), while the CTR group received a ration that provided 1.8% M starting 10 days before AI. Different superscripts indicate statistical differences (*p* < 0.05).

The mean area of the PO follicle measured on the day of AI in heifers that showed estrous behavior was 1.98 ± 0.09 cm^2^ (range 1.1–3.43 cm^2^) and was smaller (*p* = 0.0008) in NR compared to CTR heifers (NR 1.81 ± 0.1 cm^2^, *n* = 31; CTR 2.58 ± 0.19 cm^2^, *n* = 9). Among heifers that would subsequently conceive, the PO follicle was smaller (*p* = 0.016) in NR compared to CTR animals (NR 1.77 ± 0.12 cm^2^, *n* = 22; CTR 2.58 ± 0.21 cm^2^, *n* = 6; Figure 1). Also, NR heifers that failed to get pregnant (1.91 ± 0.44 cm^2^, *n* = 9) had a smaller PO follicle than pregnant CTR animals (2.58 ± 0.21 cm^2^, n = 6; *p* = 0.01) and nonpregnant CTR animals (2.57 ± 0.42 cm^2^, *n* = 3; *p* = 0.027).

In heifers that showed estrous behavior, the mean AFC on the day of AI was similar between NR and CTR heifers (NR 15.75 ± 1.023, *n* = 31; CTR 15.4 ± 1.9, *n* = 9). In heifers that would subsequently become pregnant with either a male or female calf (NR *n* = 22; CTR *n* = 6), the AFC was repeatable within the same individual on the day of AI, DG 28, and DG 55–70 (*p* = 0.66) and was similar between the NR and CTR groups during early pregnancy.

### Maternal growth performances

**Figure 2:**
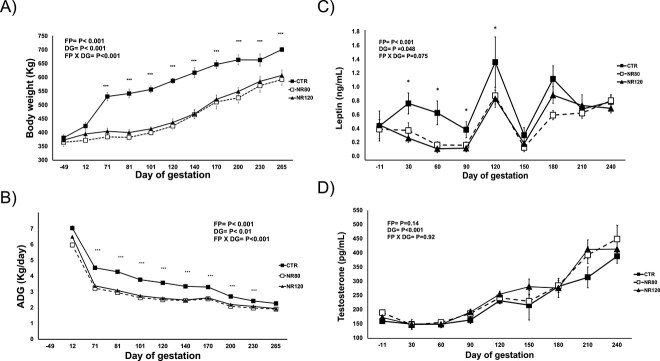
Body weight (mean ± standard error of the mean (SEM), Kg; Graph A), ADG (mean ± SEM, kg/day; Graph B), Plasmatic leptin (mean ± SEM, ng/mL; Graph C), and testosterone concentrations (mean ± SEM, pg/mL; Graph D) in dairy heifers pregnant with a single female calf (NR120, *n* = 10; NR80, *n* = 8; CTR, *n* = 5). Starting 11 days before AI, NR heifers were individually fed a ration at 0.6% of their maintenance requirement (M) up to either d80 (NR80) or d120 (NR120) of gestation, while the CTR group received a ration that provided 1.8% M. FP, feeding program; DG, day of gestation. Asterisks indicate significant differences (^*^^*^^*^p < 0.001; ^*^^*^p < 0.01; ^*^p < 0.05) between groups on a specific day of life.

Heifers that would subsequently be pregnant with a healthy single female calf in the three experimental groups (NR120 *n* = 10; NR80 *n* = 8; CTR *n* = 5) were similar in BW before the start of the differential feeding program (NR120 = 379.3 ± 14.5; NR80 = 372.9 ± 14.5; CTR = 389.6 ± 11.1 kg; Figure 2A) and BW increased as pregnancy progressed in all groups (*p* < 0.0001). Control heifers were heavier than NR80 and NR120 from day 12 of gestation to term, whereas no difference was detected between NR80 and NR120. Specifically, the differences in BW between pre-CR and DG 80 were 12 ± 8, 21 ± 7 and 163 ± 4 kg in NR80, NR120, and CTR heifers, respectively. By DG120, the mean increase in BW was 51 ± 8, 59 ± 19, and 211 ± 6 kg in NR80, NR120, and CTR group, respectively. On DG 265, dams in all groups were heavier compared to pre-CR (NR80 = 220 ± 18, NR120 = 227 ± 21, CTR = 321 ± 15 kg; Figure 2A).

The ADG was similar among groups until DG 70 (Figure 2B). From DG 71 to 230, the ADG was higher in CTR compared to NR80 and NR120 (*p* < 0.001), whereas no difference was detected between NR80 and NR120 (Figure 2, Graph B). From DG 231 to 265, the ADG was similar among groups. The mean ADG from pre-CR to DG80 was 2.95 ± 0.09, 3.08 ± 0.08, and 4.25 ± 0.09 kg/day in NR80, NR120, and CTR heifers, respectively. From pre-CR to DG120 the ADG was 2.49 ± 0.06, 2.58 ± 0.06, 3.56 ± 0.07 kg/day in NR80, NR120, and CTR heifers, respectively. The ADG from pre-CR to calving was 1.90 ± 0.07, 1.98 ± 0.04, and 2.26 ± 0.02 kg/day in NR80, NR120, and CTR heifers, respectively (Figure 2, Graph B).

### Maternal leptin and testosterone analysis

Plasmatic leptin concentrations in heifers pregnant with a single female calf (NR120 *n* = 10; NR80 *n* = 8; CTR *n* = 5) were influenced by the feeding program (FP; *p* < 0.0001) and by the DG (*p* = 0.048), whereas the interaction of both variables tended to be significant (FPxDG; *p* = 0.075). Leptin concentrations were similar among the three groups before the start of the differential feeding regime; on days 30, 90, and 120 of gestation they were greater in CTR compared to both NR80 and NR120 (*p* < 0.05), whereas no difference was detected among groups from day 150 to calving (Figure 2). Maternal circulating testosterone concentrations in pregnant dams were similar among the three experimental groups before the start of the differential feeding regime, increased as gestation progressed (DG; *p* < 0.001), but were not affected by diet (FP; *p* = 0.14) and by the interaction of both variables (FPxDG; *p* = 0.92; Figure 2).

### Calving and growth performances in calves

Single female calves born to differentially fed mothers (NR120 *n* = 9; NR80 *n* = 8; CTR, *n* = 5) had similar mean gestational length (275.92 ± 0.64 days). BW, height at withers, hip height, and back length were similar among calves in the three experimental groups from birth to slaughter at 135 days of age (Table 3).

**Table 3:** Effects of maternal feeding program in early gestation on body growth in single female calves (CTR, *n* = 5; NR120, *n* = 9; NR80, *n* = 8). FP, feeding program; Week, week of age. Data are expressed as mean ± SEM

	Days of age							FP	*P*-value Week	FP × week
Parameter	0	30	60	74	103	121	135			
Body weight, kg										
Control NR120 NR80	41.4 ± 1.1 38.3 ± 1.2 36.7 ± 0.6	49.5 ± 1.2 48.8 ± 1 48.9 ± 1.4	71.1 ± 2.7 69.9 ± 1.8 71 ± 1.3	82.2 ± 3.1 82.6 ± 2.2 85 ± 2.5	108 ± 4.3 104.1 ± 2.7 107.8 ± 4.1	110 ± 4.3 111.5 ± 5.3 108.9 ± 7.3	118 ± 3.4 119.3 ± 3.3 113.8 ± 6.7	0.68	<0.0001	0.997
Thoracic circumference, cm										
Control NR120 NR80	77.4 ± 1.2 73.9 ± 0.8 74 ± 0.9	82.4 ± 0.9 80.9 ± 1.2 80.6 ± 0.5	93.2 ± 1.5 91.8 ± 0.7 93.2 ± 0.6	100.6 ± 1.3 99 ± 0.9 99.6 ± 1.1	106.6 ± 1.3 105.1 ± 1.1 106 ± 1.4	106 ± 2.2 107.6 ± 1.4 109 ± 3.3	110.4 ± 1.8 110.7 ± 1.6 108.6 ± 2.1	0.27	<0.0001	0.875
Height at withers, cm										
Control NR120 NR80	73.6 ± 1.2 72.4 ± 0.8 72.2 ± 1.1	78.8 ± 0.5 76.6 ± 0.7 76.8 ± 0.7	85.8 ± 0.9 84.8 ± 0.5 87.1 ± 0.8	88 ± 0.6 87.9 ± 0.7 87.9 ± 0.9	96.2 ± 0.4 93.3 ± 0.6 93.6 ± 0.8	97.8 ± 0.5 96.2 ± 0.5 96.6 ± 0.7	100.6 ± 0.9 99.7 ± 1.1 99.9 ± 1.1	0.229	<0.0001	0.641
Hip height, cm										
Control NR120 NR80	79.2 ± 0.9 75.6 ± 1.0 75.5 ± 1.1	82.8 ± 0.4 81.6 ± 0.6 80.5 ± 1.2	90.6 ± 0.7 87.7 ± 0.8 87.9 ± 1.2	93.2 ± 0.4 93.1 ± 0.6 92.4 ± 0.9	100 ± 0.7 98.2 ± 0.6 98.1 ± 0.6	101.2 ± 0.7 101 ± 0.7 101 ± 0.6	105.4 ± 0.5 104.7 ± 1.2 104.4 ± 1.3	0.264	<0.0001	0.329
Back length, cm										
Control NR120 NR80	- - -	61.8 ± 1 63.6 ± 0.9 61.9 ± 0.8	68.4 ± 0.5 68 ± 0.7 68.1 ± 0.5	76.2 ± 0.9 74.9 ± 0.5 74.1 ± 1.4	85 ± 0.7 85.7 ± 0.5 85.4 ± 0.9	86 ± 1.2 87.2 ± 1.2 6.9 ± 1.0	87.6 ± 1.4 90.2 ± 0.8 88.9 ± 0.8	0.279	<0.0001	0.687

### Arterial blood pressure and echocardiography

**Figure 3:**
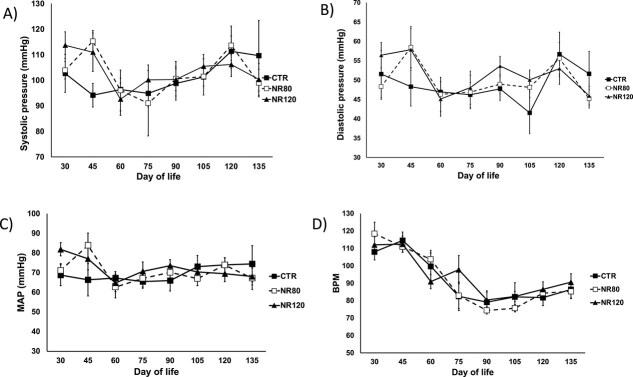
Mean resting peripheral arterial BP (mmHg) and HR (BPM) measured every two weeks from 30 to 135 days of age in female calves born to heifers exposed to a different feeding program in early gestation (NR120 = 9; NR80 = 7; CTR = 5). The Nutrient-restricted (NR) dams were fed with a ration at 0.6% of their maintenance requirement (M) until day 80 (NR80) or 120 (NR120) of gestation, whereas CTR dams received a ration that provided 1.8% M. Results are expressed as Mean ± SEM. Systolic pressure (Graph A), diastolic (Graph B), mean arterial pressure (MAP; Graph C), beats per minute (BPM, Graph D).

Twenty-one calves (NR120 *n* = 9; NR80 *n* = 7; CTR *n* = 5) underwent echocardiographic examination. One NR 80 calf was excluded from the cardiovascular evaluation and BP measurement because of echocardiographic evidence of severe ventricular septal defect. Echocardiographic variables did not differ between groups at 35 and 100 days of age; moreover, echocardiographic parameters were not influenced by the maternal feeding program (Table 4). Systolic, diastolic, and mean arterial pressure and HR (measured during arterial BP evaluation) were similar in calves in the three groups from 30 days old to 4 months of age (Figure 3).

**Table 4:** Echocardiographic parameters (mean ± SEM;) evaluated on day 35 (panel A) and 100 of life in single female calves born to dairy heifers exposed to a different feeding program in early gestation (NR120, *n* = 9; NR80, *n* = 7; CTR, *n* = 5).The NR dams were fed with a ration at 0.6% of their maintenance requirement (M) until day 80 (NR80) or 120 (NR120) of gestation, whereas CTR dams received a ration that provided 1.8% M. Results are expressed as Mean ± SEM. IVSd, interventricular septum in diastole; LVIDd, left ventricular internal diameter in diastole; LVFPd, left ventricular posterior wall in diastole; IVSs, interventricular septum in systole; LVIDs, left ventricular internal diameter in systole; LVFPs, left ventricular posterior wall in systole; HR, heart rate; FS, fractional shortening (calculated by measuring the percentage change in left ventricular diameter during systole); Aot, Aortic root in transversal section (early diastole)

Age (day)	Group	IVSd (cm)	LVIDd (cm)	LVFPd (cm)	IVSs (cm)	LVIDs (cm)	LVFPs (cm)	AOT (cm)	HR (bpm)	FS (%)
35	NR120	1.11 ± 0.17	4.28 ± 0.36	1.02 ± 0.12	1.45 ± 0.15	2.64 ± 0.3	1.76 ± 0.19	2.84 ± 0.29	115.3 ± 6.5	38.2 ± 1.9
	NR80	1.07 ± 0.06	4.42 ± 0.48	0.94 ± 0.1	1.4 ± 0.13	2.91 ± 0.4	1.55 ± 0.14	2.86 ± 0.15	114.8 ± 6.8	34.2 ± 2.1
	CTR	1.12 ± 0.05	4.76 ± 0.35	1.02 ± 0.02	1.38 ± 0.15	3.23 ± 0.4	1.6 ± 0.16	3.02 ± 0.26	116.2 ± 6	32.4 ± 2
100	NR120	1.13 ± 0.03	5.18 ± 0.12	1.01 ± 0.12	1.58 ± 0.03	3.18 ± 0.1	1.91 ± 0.9	3.55 ± 0.08	78.33 ± 2.3	38.55 ± 2.2
	NR80	1.14 ± 0.04	5.36 ± 0.15	0.99 ± 0.09	1.57 ± 0.1	3.32 ± 0.1	1.82 ± 0.06	3.55 ± 0.07	71.42 ± 4.2	37.14 ± 2
	CTR	1.11 ± 0.05	5.42 ± 0.25	1.032 ± 0.07	1.53 ± 0.1	3.42 ± 0.3	1.85 ± 0.05	3.71 ± 0.1	82.2 ± 5.9	37.2 ± 3.8

### AMH analysis in female calves

**Figure 4:**
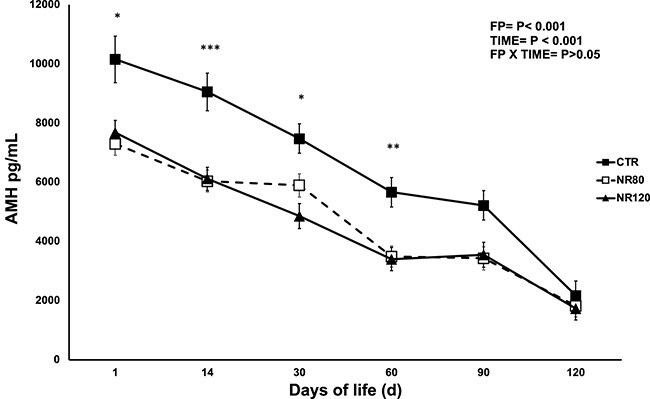
Serum AMH; mean ± SEM concentrations in female calves born to heifers exposed to a different feeding program in early gestation (CTR, *n* = 5; NR120, *n* = 9; NR80, *n* = 8). The NR heifers were fed with a ration at 0.6% of their maintenance requirement (M) until day 80 (NR80) or 120 (NR120) of gestation, whereas CTR dams received a ration that provided 1.8% M. Results are expressed as Mean ± SEM. FP, feeding program; TIME, day of life; FPxTIME, interaction between feeding program and time. Asterisks indicate significant differences (^*^^*^^*^p < 0.001; ^*^^*^p < 0.01; ^*^p < 0.05) between groups on a specific day of life.

Circulating AMH concentrations (pg/mL; Figure 4) in female calves born to differentially fed heifers (NR120 = 9; NR80 = 8; CTR = 5) were influenced by maternal feeding program (FP; *p* < 0.001) and by the age of the calf (*p* < 0.001), whereas they were not conditioned by the interaction of both variables (FPxage; *p* > 0.05). Serum AMH concentrations were greater in CTR vs both NR80 and NR120 calves at birth (*p* < 0.05), on day 14 (*p* < 0.001), 30 (*p* < 0.05), and on day 60 of age (*p* < 0.01). No difference was detected among the three experimental groups on day 90 and 120 (*p* > 0.05, Figure 4).

### Post-mortem results

Calves in the three experimental groups were similar in age (NR120 136.6 ± 3.3; NR80 136 ± 2.3; CTR 133.4 ± 3.6 day) and BW at slaughter (NR120 119.4 ± 3.3 kg; NR80 113.8 ± 6.7; CTR 118 ± 3.49.

**Figure 5:**
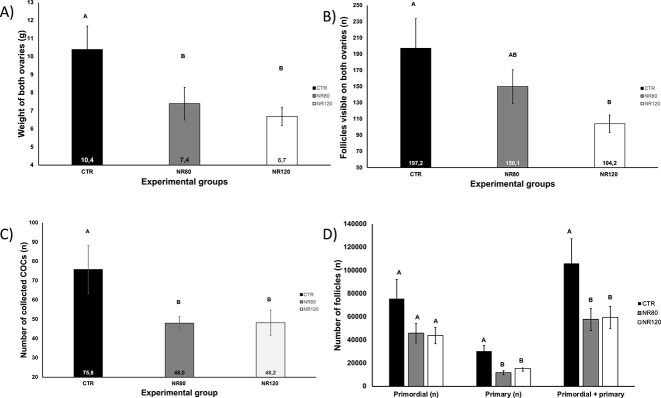
Ovarian weight (Graph A), number of visible antral follicles (Graph B), number of collected COCs (Graph C), and number of primordial and primary follicles (Graph D) in female calves born to mothers fed with differential diets in early gestation (CTR; NR until day 80 of gestation; NR80; NR until day 120 of gestation, NR120). Ovaries were retrieved when calves were 135 days old (NR120, *n* = 9; NR80, *n* = 8; CTR = 5). Results are expressed as mean ± SEM. Different superscripts indicate statistical differences (A vs B; *p* < 0.05).

Pairs of ovaries collected from NR120 calves were lighter compared to gonads retrieved from CTR individuals (*p* < 0.05); also, pairs of ovaries in the NR80 group tended to be lighter than those from CTR animals (*p* = 0.07), whereas ovarian weight was similar between NR120 and NR80 (Figure 5, Graph A). No correlation was detected between weight of the ovaries and BW at slaughter in female calves (*R* = 0.05; *p* < 0.05).

As reported in Figure 5 (Graph B), the total number of visible antral follicles on the ovarian surface was different among groups (*p* = 0.037). Calves born to NR120 mothers had less visible antral follicles compared to CTR calves (*p* < 0.05), whereas no difference was detected between NR80 and NR120 and between CTR and NR80 calves, respectively. The mean number of visible antral follicles was positively correlated to ovarian weight (*R* = 0.49; *p* < 0.05).

The mean number of COCs (cumulus oocytes complexes) retrieved per animal was different among groups (*p* = 0.029). Less COCs/individual were collected from NR80 and NR120 calves as compared to CTR individuals (*p* < 0.05), but no difference was detected between NR80 and NR120 calves (Figure 5, Graph C). The mean number of retrieved COCs was positively correlated to ovarian weight (*R* = 0.65; *p* < 0.05).

The number of primordial follicles was similar among three experimental groups, whereas CTR calves had more primary follicles compared to NR 80 and NR120 animals (*p* < 0.001; Figure 5, Graph D). No difference in the number of primary follicles was detected between NR120 and NR80 individuals (Figure 5, Graph D). The mean number of primordial and primary follicles were positively correlated to ovarian weight (*R* = 0.73; *p* < 0.01 and *R* = 0.56; *p* < 0.01, respectively).

Heart weight (CTR = 624 ± 0.02; NR80 = 558 ± 0.03; NR120 = 598 ± 0.03 g), internal diameter of the aorta (CTR = 2.1 ± 0.1; NR80 = 2.1 ± 1.8; NR120 = 1.8 ± 0.2 cm), and circumference of the aorta (CTR = 9 ± 0.8; NR80 = 9.4 ± 0.4; NR120 = 9.6 ± 0.5 cm) were similar among all calves. Heart weight and BW at slaughter were positively correlated (*R* = 0.77; *p* = 0.0001). Weight of both kidneys was similar between the three different experimental groups (CTR = 497.6 ± 14.4; NR80 = 458.6 ± 17.6; NR120 = 503.6 ± 29.3 g), and the weight of both kidneys and BW at slaughter were not correlated (*R* = 0.37; *p* = 0.09).

## Discussion

The main findings of this study are that (i) maternal energy restriction from pre-CR to either day 80 or 120 of gestation in dairy heifers reduced the size of the PO, increased peripheral leptin serum concentrations without altering testosterone levels during pregnancy in the dams; (ii) daughters of nutritionally restricted mothers had impaired ovarian development, as assessed by reduced gonadal weight, fewer surface antral follicles, primary follicles, and recovered COCs, as well as lower circulating AMH concentrations, but (iii) similar body weight, cardio-vascular morphology and function compared to the progeny of control-fed mothers, as assessed by peripheral arterial BP, echocardiography, *post-mortem* heart weight, and aortic circumference.

For the first time, we provide evidence that nutrient restriction from 10 days before CR to day 80 of pregnancy can impair ovarian development in bovine progeny, indicating that a sub-optimal *in utero* environment for the first 2.6 months of gestation can negatively influence folliculogenesis in female offspring. When calves were 4.5 months old, ovaries collected from NR80 animals had fewer primary follicles, and fewer COCs/individuals were retrieved from NR80, as compared to CTR individuals. Also, ovaries collected from NR80 calves tended to be lighter than gonads retrieved from CTR calves. In line with our observations (ovarian weight vs. primordial follicles *R* = 0.73, *p* < 0.01; primary follicles *R* = 0.56, *p* < 0.01; visible antral follicles *R* = 0.49, *p* < 0.05; retrieved COCs *R* = 0.65, *p* < 0.05) a positive correlation between ovarian weight and the number of ovarian follicles, both small (*r* = 0.64; *p* < 0.0001) and medium follicles (*r* = 0.46; *p* < 0.001), was previously established in cattle [61]. In addition, calves born to NR80 dams had lower circulating AMH concentrations from birth to day 60 of life compared to offspring of control-fed mothers.

Such effect on female gonads was comparable to the one elicited by nutrient restriction imposed until the first 120 days of pregnancy; indeed, calves born to NR80 and NR120 heifers had similar serum AMH from birth to four months of age and similar ovarian weight, primordial and primary follicles and collected COCs/individual *post-mortem*. Such evidence indicates that the same level of energy restriction imposed for the first 2.6 months of fetal life can result in similar ovarian phenotype in juvenile offspring as when imposed for the first four months of pregnancy. Since the peak number of germ cells occurs between days 91 and 110 in cattle [11, 20, 21], nutrient restriction until day 80 may be sufficient to compromise the activation of the primordial follicular pool by influencing the most part of this critical window of development. Another plausible explanation is that when the nutritional challenge lasted until day 120 of pregnancy, a compensatory mechanism may have been activated to limit the detrimental impact of the longer nutritional challenge; indeed, maternal BW and ADG were similar between NR80 and NR120 dams throughout gestation.

Remarkably, present evidence that maternal nutrient restriction until day 120 of gestation impaired ovarian development in dairy calves is in accordance with previous results obtained in beef cattle [35]. Maternal nutritional restriction (NR, 0.6 M vs CTR, 1.2 M) from shortly before CR to day 110 of gestation impaired the size of the ovarian reserve in their daughters, as assessed by reduced numbers of antral follicles recruited per follicular wave, lower serum AMH, and greater FSH concentrations compared to offspring of control-fed dams [35]. Also, heifers born to dams that received a low-protein diet during the first trimester followed by a high-protein diet during the second trimester of pregnancy had smaller-sized largest ovarian follicles at puberty and lower densities of primordial and primary follicles and healthy antral follicles as adults [62]. Beef and dairy cattle have been extensively selected to develop different phenotypic characteristics, and thus, their metabolism during gestation may be different. Nonetheless, evidence supporting the negative impact of nutritional imbalance in the first trimester of gestation is reported in both bovine breeds.

Moreover, reductions in the total number of ovarian follicles and alterations in ovarian function of the progeny have been reported consistently across several maternal nutrient restriction models in different species [63]. Women exposed to famine during their fetal life underwent menopause at an early age [64] and had increased rates of sterility [65], potentially due to a limited ovarian reserve. Intrauterine growth restriction is associated with reduced primordial follicle development and premature follicle loss in female neonates [66]; further, reduced ovarian volume (an indirect measure of ovarian reserve) has been reported in women exposed to prenatal growth restriction or born small for gestational age [67, 68]. Similarly, in rodents, studies using maternal low-protein diets, caloric restriction, and starvation experimental models report decreases in total follicle number in female offspring [63]. Taken together, these findings indicate that fetal germ cells/oocytes and follicles are highly vulnerable to imbalanced nutrition in mammals, with potential long-term consequences on fertility.

In the present study, when calves were 4.5 months old, the number of primordial follicles was numerically reduced in offspring of both nutritionally restricted groups compared to control, but the number of primary follicles was significantly lower in NR80 and NR120 daughters compared to CTR progeny. This could be indicative of a delay in follicular activation in prepubertal calves exposed to moderate energy imbalance as embryos/fetuses compared to age-matched animals exposed to adequate nutrition. Taken together, these findings indicate that moderate nutritional restriction from pre-CR to either day 80 or 120 of gestation may impair ovarian development in the progeny, with similar impacts regardless of the duration of the energy imbalance. Interestingly, in agreement with the present findings, the reduction in total number of follicles occurred consistently across different nutritional models, yet the follicular class that was most affected varied across studies depending on the timing, duration and severity of energy restriction [63]. Decreased primordial follicles were observed in offspring of mothers undernourished during gestation in bovine [62] and murine species [69]; maternal protein restriction from pre-CR to lactation in rats depleted primordial follicles numbers by 37% in juvenile and 51% in adult progeny, respectively, yet it had no effects on other follicle classes [70]. In a rodent model of an isocaloric, low protein diet during gestation, followed by suckling of control-fed dams to induce BW catch-up growth in the offspring, primordial follicle density was reduced in 6-month-old progeny and up-regulation in ovarian oxidative stress was reported [71]. Similarly, rats born to undernourished mothers during gestation had lower numbers of antral follicles and a three-fold increase in ovarian protein carbonyl levels, which are considered markers of protein oxidation [72]. Thus, altered oxidative stress conditions have been proposed as a potential biological pathway that links unbalanced maternal nutrition and developmental programming of the size of the ovarian reserve in the progeny, but differences in the timing of ovarian development should be considered when comparing across species and diverse experimental models.

Peripheral AMH concentrations are positively correlated (*r* > 0.90) with the total number of morphologically healthy follicles and oocytes in ovaries of young adult cattle [31]. In the current study, AMH serum concentrations were, on average, 27–44% greater from birth to three months of age in calves born to control dams compared to both nutrient-restricted groups, providing evidence for an impairment of the formation of secondary and early antral follicles. Nonetheless, no difference was observed in AMH concentrations when calves were four months old. Wave of antral follicular growth occurs in calves for 2 weeks of age and number of follicles recruited/wave increases from 2 to 14 weeks of age [73], thus variations in AMH concentrations observed before puberty may be reflective of changes in growth patterns of small antral follicles.

Albeit evidence indicates that AMH levels are stable during the bovine estrous cycle and are repeatable in the same individual across different estrous cycles [30, 32, 74, 75], variations in AMH levels in prepubertal animals were previously observed. Concentrations of AMH increased during the first 2 months of age, decreased at 5, and were stable at 8–9 months of age, around the time of first ovulation in beef and dairy calves [76]. In Maine–Anjou beef heifers, plasma AMH concentrations were found to increase rapidly between 1 and 3 months of age, to remain high at 6, and to decline slowly until 12 months of age, which corresponds to the age of ovulation for this breed [77].

In Japanese Black heifers, AMH concentrations gradually increased from birth to peak at week 10 of age (2.3 months) and declined steadily [78]. On the contrary, here we observed a constant decline in AMH peripheral concentrations from birth to four months of age in all calves, regardless of maternal dietary regimen. Recently, the mean peripheral AMH concentrations in 60-day-old dairy calves (*n* = 149) were estimated as 1045.91 ± 976.21 pg/mL [79]; in the present study the mean peripheral AMH concentrations at the same age were 3889.94 ± 1272.50 pg/mL, whereas in Japanese Black heifers (*n* = 6), serum AMH levels at 2.3 months of age were 0.33 ± 0.1 ng/mL [78]. Circulating AMH concentrations were similar between prepubertal rats born to 50% calorie-restricted mothers and control-fed dams, but the same level of maternal undernutrition decreased serum AMH levels in young adult offspring [80]. Taken together, these observations provide evidence for a detrimental impact of maternal energy restriction on AMH circulating levels in female progeny, but imply that AMH secretion and metabolism pattern in prepubertal animals have yet to be completely elucidated.

To the best of our knowledge, this is the first study in which echocardiography has been used to investigate developmental programming of the cardiovascular system in calves. Cardiovascular system in the progeny was not influenced by maternal diet, as echocardiographic parameters, systemic arterial BP, *post-mortem* heart weight, and aortic circumference were similar among groups. These findings are in contrast with results obtained in beef cows, where maternal undernutrition determined an increase in arterial BP in female offspring and an enlargement of the aortic trunk [35]. Such discrepancy could be due to an age difference: in the present study, calves were studied until they were 4.5 months of age, whereas beef calves were monitored until they were 22 months old [35]. It is plausible that a difference in cardiovascular function could be detectable in a longer timeframe. In mice and humans, the impact of maternal nutrition on the cardiovascular system of the progeny is evident in adulthood. Adult mice born to undernourished mothers have lower resistance to physical activity, reduced left ventricular mass, and a thinner LVFPd compared to offspring control dams [81]. Heightened BP was reported in 40-year-old men born to mothers who consumed a low-protein diet during their pregnancy [82]. Adult women (46 years old) born during the Chinese famine had an increased risk of developing hypertension in adulthood [83]. Moreover, early onset menopause increased the risk of heart disease and stroke in women and was associated with low birth weight in childhood [84, 85].

In cattle, maternal protein restriction (7%CP vs 14% CP) from 60 days prior to CR to 23 days post-CR resulted in lower heart weight in 98-day-old female fetuses, increased HR in female calves at birth with similar birth weight, and decreased systolic BP in female calves at six months of age, with no effect at 8 or 10 months [86]. In that experiment, maternal pre-CR exposure to protein restriction lasted two months, whereas, in our work, heifers were exposed to a nutritionally restricted diet 10 days before insemination. Bovine follicular development from the primary to the ovulary stage is estimated to last 90 days [87]; thus, a longer pre-CR exposure to nutritional imbalance may have exerted a significant programming effect on the cardiovascular function of the progeny. Further, in the present work, nutrient-restricted heifers were exposed to reduced caloric intake with 14.5% CP, whereas in the investigation from Hernandez et al. [86], diets were isocaloric and the low protein diet provided 7% CP. The differences in dietary treatment (protein versus total nutrients limitation) together, with the disparity in the window of exposure to the nutritional imbalance, may explain these apparently conflicting findings. Finally, it should be noted that BP is remarkably labile; thus, when BP is assessed with non-invasive techniques in humans several methodological procedures are recommended and invasive arterial BP measurement is the gold standard in intensive therapy units [88]. Albeit we previously validated the tail-cuff method in cattle [35] and methodological consistency was guaranteed during BP measurement, the technical limits of this technique in cattle should be considered and may underpin the inconsistent findings among reports.

In the present study, birth weight was not influenced by maternal diet; also, BW and several biometric measures in the progeny were similar among experimental groups until slaughter. These findings may be explained by the fact that fetal energy requirements are low during the first two trimesters of gestation in cattle, whereas they increase steeply from day 190 to term [89]. Postnatal development can program cardiovascular health, as babies born small for gestational age, or with low birth weight, and having a rapid postnatal catch-up growth have increased risk of cardiometabolic pathologies [90, 91]. In our study, the lack of impact of maternal diet on the progeny’s cardiovascular system could be explained by the similarity in birth weight and postnatal growth among calves. Murine offspring exposed to a maternal low protein diet and fed a non “growth diet” to ensure that they did not undergo catch-up growth after weaning remained smaller than control offspring but did not exhibit adverse cardiovascular health outcomes up to 1 year of age [91]. Nonetheless, more investigations in nonmurine mammals on the potential early determinants of cardiovascular health are required.

Energy restrictions imposed 10 days before AI resulted in smaller PO, but CR (28 days post-AI) and PR (60 days post-AI) were unaltered. Follicular size has been positively associated with the acquisition of developmental competence by the oocyte in different species. Oocytes retrieved from large follicles had greater developmental competence in sheep [92], pig [93], mare [94], and cattle [95, 96] compared to oocytes obtained from smaller follicles. It is estimated that follicular development from primordial to ovulatory stage takes 4–6 months. in cattle and that follicular growth from the preantral to preovulatory phase occurs in approximately 90 days [87] and numerous studies analyzed the effect of malnutrition on the developmental dynamics of ovarian follicles in cattle. Long-term moderate dietary restriction can lead to a reduction in the growth rate of the dominant follicle, its maximum diameter, and duration [97]. For example, in beef cattle fed with diets at various levels of restriction (0.7; 1.1 or 1.8% of live weight as a daily dry matter) for 5 weeks, the maximum diameter and persistence of the dominant follicle were reduced even reaching ovulation [98]. Acute energy restriction (1.2–0.4 M), starting the day before the end of the heat synchronization protocol, caused a significant reduction in the growth rate and the maximum diameter of the dominant follicle in the first follicular growth wave of the subsequent estrous cycle [99]. Present evidence indicates that in dairy nulliparous heifers, acute moderate energy restriction in the periovulatory period can limit the growth of the ovulatory follicle with no impact on the developmental competence of the ovulated oocyte. Nonetheless, the reduction in preovulatory follicle size could be reflective of a different environment of the ovulatory follicle that may have contributed to programming the observed phenotype in the progeny.

Nutrient restriction resulted in a transient decrease in leptin peripheral concentrations, a hormone that is primarily secreted by white adipose tissue and is positively correlated with body fat [100]. In growing adolescent ewes, undernutrition up to 50- or 100-days gestation during pregnancy reduced leptin concentrations [101]. Acute fasting decreased plasma concentrations of leptin in mature beef cows [102] and pregnant cows fed to obesity during the second and third trimester of gestation exhibited greater plasma leptin concentrations than dams fed to maintain a moderate body condition or fed a restricted diet to achieve a very thin body condition [103]. In the same experiment, reduced expression of a short leptin receptor variant *(ObRc)* in the choroid plexus of the brain was observed in offspring born to dams with very thin compared to obese and moderate body condition, but the ovaries were not examined [103]. Evidence indicates that leptin has a stimulatory effect on GnRH secretion [104] and that pituitary gonadotropes bear leptin receptors [105]. Reduced leptin concentrations in nutrient-restricted dams may have programmed the fetal neuroendocrine reproductive axis, indirectly leading to the observed impairment of the size of the ovarian reserve in juvenile progeny. On the other hand, since all ovarian follicular cells have leptin receptors [105], maternal leptin may have directly programmed the establishment of the follicular pool in the fetal ovary. Indeed, physiological levels of leptin stimulate granulosa and theca cells and, in partnership with growth factors, GH and FH promote follicular development to the antral stage [105].

Peripheral leptin levels regulate testosterone production by modulating the secretion of GnRH [47] and resulting in a negative correlation between the two hormones in cattle [35] and humans [106]. Here, this pattern was not observed; indeed, testosterone levels during gestation were similar among the experimental groups. This finding was also in contrast with our previous results in beef heifers, as nutrient-restricted dams had increased testosterone serum concentrations [35]. Here, a constant increase in testosterone levels as pregnancy progressed was evidenced in all pregnant heifers. Similar patterns were previously reported with gradual increase and approximately double from pre-CR to the third trimester [107–109]. In Holstein heifers carrying a female fetus, testosterone concentrations increased from 4 to 7 months of pregnancy [108], and peripheral concentrations were similar to the ones reported here. Thus, we can speculate that testosterone peripheral concentrations were within physiological ranges and that energy imbalance did not alter testosterone production.

Our study shows that moderate energy restriction in dairy heifers has deleterious effects on ovarian development of their daughters, indicating that conditions during prenatal life may contribute to the high variability in the size of the ovarian reserve observed in mammals, such as cattle. Nutritional restriction resulted in a similar impairment of the ovarian reserve in the progeny, regardless of its duration (until day 80 or 120), suggesting that the window of development that encompasses the peri-ovulatory period to the first 2.6 months of gestation is critical for ovarian development. These novel findings are relevant because several studies indicate that cattle with a low ovarian reserve have phenotypic characteristics that are associated with suboptimal fertility [15, 76]. No effect of maternal undernutrition on body growth and on the morphology and physiological function of the cardiovascular system of female offspring was assessed in the first 4.5 months of life. Albeit the negative impact on arterial BP and cardio-vascular physiology may have been evident in adulthood, as previously reported in cattle, mice, and humans [35, 81–83], the present findings contribute to increased knowledge on the factors that program cardiovascular health in humans, because they were obtained in a domestic animal model with similar gestational length as women. The mechanism whereby exposure to undernutrition *in utero* may program gonadal development remains largely unidentified, as we did not observe a transient increase in maternal testosterone; nonetheless, the potential role of maternal leptin may be further explored in future studies.

## Data Availability

The data underlying this article will be shared on reasonable request to the corresponding author.
